# Self-Assembled (Nano)Structures of Human Serum Albumin with Thermoresponsive Chitosan-*g*-PNIPAM Graft Copolymer

**DOI:** 10.3390/polym18040515

**Published:** 2026-02-19

**Authors:** Florin Bucatariu, Larisa-Maria Petrila, Timeea-Anastasia Ciobanu, Marius-Mihai Zaharia, Stergios Pispas, Marcela Mihai

**Affiliations:** 1 Petru Poni Institute of Macromolecular Chemistry, 41A Grigore Ghica Voda Alley, 700487 Iasi, Romania; fbucatariu@icmpp.ro (F.B.); larisa.petrila@icmpp.ro (L.-M.P.); timeea.ciobanu@icmpp.ro (T.-A.C.); zaharia.marius@icmpp.ro (M.-M.Z.); pispas@eie.gr (S.P.); 2 Theoretical and Physical Chemistry Institute, National Hellenic Research Foundation, 48 Vassileos Constantinou Ave., 116 35 Athens, Greece

**Keywords:** chitosan, human serum albumin, nanostructures, thermoresponsive

## Abstract

Protein–polyelectrolyte entities (complex, coacervates, flocs, gels, etc.) are of great interest due to their potential applications in biological and medical fields. This study focuses on investigating the interactions between a model protein, human serum albumin (HSA) and a newly synthesized hybrid thermoresponsive copolymer based on chitosan polysaccharide grafted with poly(*N*-isopropylacrylamide) synthetic polymer chains (Chit-*g*-PNIPAM), in aqueous media, by mixing the individual component aqueous solutions. Depending on the mixing molar ratio and the order of addition of the two components (protein and copolymer), either stable nanostructured suspension or macrostructures’ phase separation have been observed. Dynamic light scattering (DLS) results reveal that the Chit-g-PNIPAM/HSA_x_ (molar ratio 5:x, where x = 1, 2, 3, 5, 10 and 15) nanostructures’ and HSA/Chit-g-PNIPAM_x_ (molar ratio 100:x, where x = 1, 2, 3, 10, 20, 30, 40 and 50) structures’ formation depend on the molar ratio of the two components as well as on the order of addition, with first component amount being kept constant in aqueous solution and second component solution added drop-by-drop in the solution of the first component. Additional information regarding the thermoresponsiveness and stability vs time of the formed (nano)structures were acquired using turbidimetry and DLS measurements.

## 1. Introduction

The interaction between oppositely charged macromolecules has attracted significant importance in the field of soft matter, as the assembly of such compounds enables the formation of organized (nano)structures with a broad spectrum of applications. Such macromolecular compounds include synthetic polymers, polysaccharides, nucleic acids, and proteins, which can serve as building blocks for the assembly of interpolyelectrolyte complexes or coacervates [1,2]. The association of macromolecules is mainly the result of electrostatic interactions between their ionized functional groups. However, an important role is played by hydrogen bonding and hydrophobic interactions, depending on the assembly conditions, which favor the interaction and stabilization of (nano)structures [3]. Various parameters, including the ionic strength, pH, temperature, concentration, or surface charge, influence the assembly behavior of charged macromolecules [4,5,6,7,8,9,10,11,12]. Their understanding and control allow the design of complex nanostructures with tailored properties. Eremenko et al. [12] used microwave dielectrometry to study the complex permittivity parameters under small changes of HSA concentration in aqueous solution in the presence of different amounts of NaCl and glucose.

The importance of such (nano)structures resides in their applications in emerging fields spanning from drug delivery [13,14] to tissue engineering [15,16], food chemistry [17,18] or environmental protection [19,20]. The formation of (nano)structures by the interaction between proteins and polysaccharides is of special interest to the biomedical field, finding potential applications in drug delivery [21] or tissue engineering [22].

One of the most important polysaccharides is chitosan, a linear polymer obtained by the alkaline deacetylation of chitin, a natural macromolecule found in the exoskeleton of crustaceans such as lobster, shrimp, or crab [23]. From the structural point of view, chitosan is formed by randomly distributed β-(1 → 4)-linked D-glucosamine and N-acetyl-D-glucosamine units [24]. The solubility and charge distribution of chitosan are strongly correlated with its molar mass, the degree of deacetylation of the macromolecular chain and the deacetylation/reacetylation processes. Chitosan has a net positive charge in acidic media due to the protonation of its primary amino groups. The presence of ionizable functional groups on the chitosan backbone enables its interaction with negatively charged biomolecules, forming electrostatic complexes.

From an application perspective, chitosan is widely recognized as an easily available, biocompatible, and biodegradable polysaccharide, finding important applications in the biomedical, cosmetic, and nutrition fields [25]. The properties of chitosan can additionally be improved by employing chemical reactions including acylation [26], quaternization [27], or alkylation [28]. Another interesting approach in this direction is the grafting of synthetic polymeric chains onto the chitosan backbone, a method that enables the introduction of additional functionalities, such as the pH or temperature response. For example, the grafting of poly(*N*-isopropylacrylamide) (PNIPAM) can impart chitosan with thermoresponsive behavior. PNIPAM is a synthetic polymer with a distinct structure consisting of hydrophilic amide and hydrophobic isopropyl groups. The thermoresponsive behavior of this polymer is exhibited around the lower critical solution temperature (LCST ~32 °C) when the polymeric chains pass through a reversible transition from soluble hydrated to an insoluble dehydrated state [29]. The increase in temperature is generally associated with the disruption of hydrogen bonds and an increase in hydrophobic interactions between the isopropyl groups [30]. This transition is accompanied by an increase in the turbidity of the system, and the thermoresponsive behavior is generally conserved upon the grafting of PNIPAM chains to polysaccharide chains, including Chit [31]. LCST behavior is reminiscent of aspects of protein solution behavior regarding globule formation, hydration, denaturation, and salting-out effects through conformation/solvation processes, and PNIPAM has long been considered as a model polymer to study protein behavior in aqueous media [32].

Human serum albumin (HSA) is the most abundant protein in human plasma, accounting for about 60% of plasma. HSA is a globular protein with a molecular weight of 66.5 kDa, comprised of 585 amino acid residues organized into three homologous α-helical domains [33]. HSA has an isoelectric point (iep) of about 4.7, being positively charged at pH values below the iep and negatively charged above it. The coexistence of positively and negatively charged amino acid residues on the HSA molecule allows it to interact with charged compounds such as synthetic or natural polymers. Moreover, HSA presents hydrophobic amino acid residues that can be involved in the formation of hydrophobic and hydrogen bonding interactions, respectively [34], making HSA an excellent candidate for the formation of complexes with oppositely charged compounds. HSA plays a vital physiological role in the maintenance of osmotic pressure as well as in the transportation of various molecules, including hormones, fatty acids, and other metabolites, as a biomarker for various diseases including cancer or diabetes [35,36], as well as for drug delivery systems [37,38,39,40] and tissue engineering [41,42]. Its high biocompatibility, biodegradability, and well-characterized structure make it an attractive component for the design of hybrid nanostructured systems intended for biomedical applications. There are many potential applications of protein–polymer complexes, beyond their established roles in controlled drug delivery, which involved interactions between modified polymers and HSA, such as protein-triggered biosensing and diagnostics [43,44], smart injectable hydrogels [45], targeted cancer therapy and tumor microenvironment engineering [46,47], protein stabilization [48], antifouling and biointerface engineering [49,50], tissue engineering and cell culture systems [51] and environmental biotechnology [52].

Previous studies have reported the synthesis and thermoresponsive properties of chitosan-*g*-PNIPAM graft copolymers. For example, Bao et al. [53] investigated the LCST behavior of chitosan grafted with PNIPAM at varying grafting densities, demonstrating tunable phase transition temperatures in response to environmental conditions. Similarly, Babelyte et al. [54] explored the effect of copolymer composition on the sol–gel transition of such graft copolymers in aqueous media. Our previous study [31] described the grafting procedure of an RAFT-obtained PNIPAM on chitosan (which contains one PNIPAM chain to about 70 chitosan structural units) and evidenced the copolymer thermoresponsive behavior. The present study focuses mainly on a novel preparation route of protein–copolymer complexes and a detailed analysis of how the molar ratio between components and the order of addition could influence the thermoresponsive behavior and the particle size of the formed (nano)structures, extending our previous findings [31]. These aspects have not been fully addressed in the prior literature and represent the novel contributions of the current manuscript. Thus, this study presents the interaction of HSA, as a model plasma protein, and a custom-made copolymer obtained by the grafting of thermoresponsive PNIPAM chains onto the water-soluble chitosan chain, towards the formation of Chit-g-PNIPAM/HSA and HSA/Chit-g-PNIPAM (nano)structures. The current investigation focuses mainly on the basic understanding of the assembly process and ways of controlling it under various conditions—mixing protocol, ratio of the components—as well as the behavior of the obtained self-assembled (nano)structures under changes in the solution temperature. The results and relevant discussion emphasize the role of electrostatic and hydrophobic interactions and of hydrogen bonding in the formation of complex protein–polymer (nano)structures of various sizes and their properties in aqueous media that would be interesting and useful for biomaterials development.

## 2. Materials and Methods

### 2.1. Materials

The water-soluble copolymer, Chit-*g*-PNIPAM (M_w_ = 206,800 g/mol, 19.9% PNIPAM and 80.1% Chit, 1 PNIPAM/70 chitosan structural units), was synthetized in our laboratory [31]. Human serum albumin (HSA) (M = 66,000 g/mol) and phosphate buffer saline (PBS) were purchased from Sigma-Aldrich (Sigma Chemical Co.; St. Louis, MO, USA). All aqueous solutions were prepared in Millipore water from an EVOQUA Ultra Clear TPTWF (Evoqua Water Technologies LLC; Barsbüttel, Germany).

### 2.2. Characterization Methods

Cloud point (CP) analysis was used to establish the LCST of the Chit-*g*-PNIPAM copolymer. The LCSTs of aqueous solutions (1.2 mg/mL and 3.2 mg/mL) were obtained using a LOVIbond TB300 IR turbidimeter (Tintometer GmbH Lovibond^®^; Dortmund, Germany), with the LCST being estimated from the variation in optical density (OD) associated with copolymer phase separation. The change in OD was measured as a function of the temperature, from 60 to 25 °C. The heating of the Chit-*g*-PNIPAM solution was conducted in a water bath, and then, the turbidity was measured during cooling from 60 to 25 °C (Figure S1, Supplementary Materials).

Rheology measurements have been carried out with a high-precision MCR 92 Rheometer (Anton Paar, Graz, Austria), which is a modular and versatile instrument designed for advanced rheological analysis, with a cone–plane geometry (the upper plate with a 25 mm diameter and a gap of 207 μm) equipped with a Peltier system for temperature control. The rheometer supports rotational speeds from 0.001 rpm to a maximum of 1500 rpm, providing a torque range up to 125 mN·m. In rotational mode, it achieves a minimum torque of 1 μN·m with a resolution of 100 nN·m. The rheological tests for the copolymer solution (C_Chit-g-PNIPAM_ = 10 mg/mL) were performed at temperatures ranging from 25 °C to 50 °C. The dynamic viscosity (mPa·s) curves have been obtained by measuring the relationship between the shear stress and shear rate at different temperatures.

Dynamic light scattering (DLS) measurements were used to assess the particle size diameter. The scattered light intensity of the Chit-*g*-PNIPAM/HSA and HSA/Chit-*g*-PNIPAM self-assembled (nano)structures, as well as that of the components of the complexes, have been quantified as a function of the temperature, molar ratio of complexes components, order of addition and time. DLS measurements were performed on a Litesizer DLS 500 (Anton Paar, Graz, Austria) equipped with a 40 mW semiconductor laser diode operating at a wavelength of 658 nm and at a scattering angle of 90°. The samples were measured after a 60 s equilibration period at least 3 times, with each measurement being an average of 3 runs of 10 s duration.

### 2.3. HSA Protein/Chit-g-PNIPAM Copolymer Self-Assembled (Nano)Structures’ Formation

The aqueous solutions of Chit-*g*-PNIPAM (1 mg/mL) and HSA (1 mg/mL) were prepared through copolymer and protein dissolution in Millipore water at room temperature until the solid samples were completely dissolved. Two series of Chit-*g*-PNIPAM/HSA and HSA/Chit-*g*-PNIPAM self-assembled (nano)structures were prepared using a drop-by-drop addition of one component into the solution of the second component. In the first series, HSA solution was added drop-by-drop into the solution of the Chit-*g*-PNIPAM. Therefore, Chit-*g*-PNIPAM/HSA dispersed (nano)structures were obtained, with different molar ratios between components (moles of copolymer chains relative to moles of protein, [Chit-*g*-PNIPAM]:[HSA] = 5:*x*, where *x* = 1; 2; 3….15). In the second series, Chit-*g*-PNIPAM was added drop-by-drop into the solution of HSA. Thus, a series of HSA/Chit-*g*-PNIPAM self-assembled (nano)structures were obtained at different molar ratios between components ([HSA]:[Chit-*g*-PNIPAM] = 100:x, where x = 1, 5, 10, 20, 30, 40, 50). The molar ratio between components was controlled, taking into account the weight average molar masses (M_HSA_ = 66.000 g/mol and M_Chit-g-PNIPAM_ = 204,700 g/mol) and the added volumes.

## 3. Results

**Figure 1:**
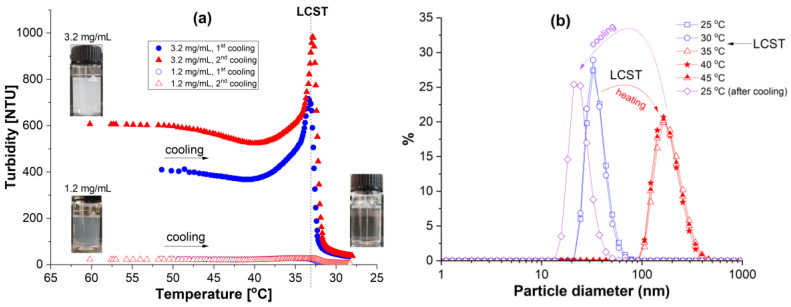
LCST determination of Chit-*g*-PNIPAM copolymer using (**a**) turbidity and (**b**) DLS (number weighted size distributions) measurements.

The copolymer Chit-g-PNIPAM was synthesized by performing free-radical “grafting to” reaction of chitosan with the active chain end of PNIPAM prepared by RAFT polymerization, as discussed in detail in our previous publication [31]. The CP and DLS measurements were used to establish the LCST of the Chit-g-PNIPAM aqueous solution (Figure 1). The LCST, associated with polymer phase separation, was estimated from the variation in the turbidity and particle number size distribution as a function of temperature in a cooling (turbidimetry) (Figure 1a) or in a heating–cooling process (DLS) (Figure 1b and Figure S1, Supplementary Materials).

The change in turbidity was measured as a function of the temperature decrease from 50 to 25 °C (Figure 1a, triangles), followed by a repeated cooling sequence from 60 to 25 °C (Figure 1a, circles) at two different copolymer concentrations (1.2 and 3.2 mg/mL). The initial turbidity, which increased with the copolymer concentration, is generated by the collapsed dense copolymer nanoparticles and followed a straight path during the cooling process, with a small decrease until 40 °C. In the range 40–33 °C, the copolymer solution with a higher concentration presented a sharp increase in the turbidity values, right before the LCST region of the system; this fact is attributed to the swelling process of the previously formed nanoparticles, right before their solubilization into individual copolymer chains. At a lower concentration of copolymer (1.2 mg/mL), this phenomenon was barely detected with the turbidimeter, probably due to the limitation of this method. The repeated measurements on the same sample, previously heated at 60 °C in a water bath, cooled and reheated, showed the same behavior around the LCST value (33 °C), demonstrating the reversibility of the Chit-*g*-PNIPAM coil-to-globule transition in aqueous solution during multiple heating–cooling processes. In addition, the Chit-*g*-PNIPAM aqueous solution (1 mg/mL) has been investigated using DLS measurements. In these studies, the particle diameter, estimated from the number weighted size distribution, is situated around 40 nm below LCST and 200 nm above LCST, most probably due to multiple Chit-*g*-PNIPAM chains involved in nano-aggregate formation above 33 °C, based on hydrophobic/H-bonding inter-chain copolymer interactions. This type of phase-separated nano-aggregates are re-dissolved below LCST during the cooling of the system in the DLS measuring cell, as can be seen in Figure 1b (number weighted size distributions) and Figure S1 (intensity weighted size distribution). At the end, at 25 °C, the particle diameter is situated at 30 nm. This temperature responsiveness of the Chit-*g*-PNIPAM copolymer, established by the turbidimetry and DLS measurements, is due mainly to the PNIPAM-grafted side-chains, which preserve the LCST of PNIPAM in the copolymer at around 33 °C.

**Figure 2:**
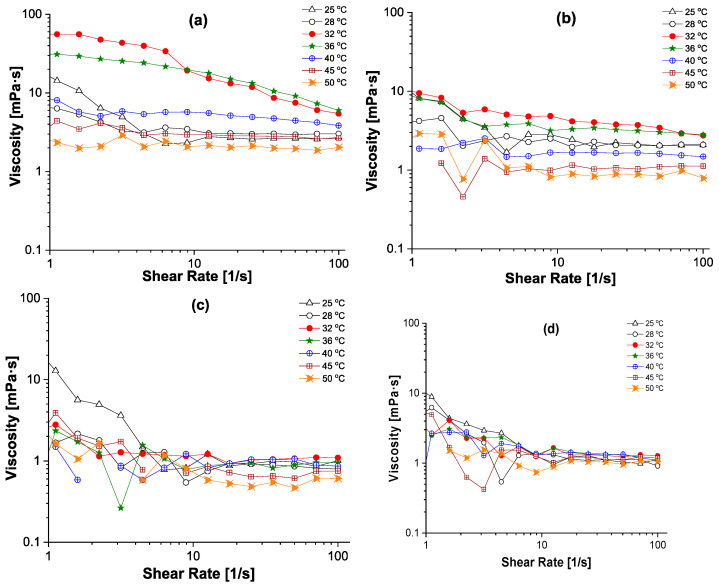
Determination of dynamic viscosity (mPa·s) of highly concentrated ((**a**) *C*_Chit-g-PNIPAM_ = 10 mg/L in water, pH = 6; (**b**) *C*_Chit-g-PNIPAM_ = 10 mg/L in PBS, pH = 7.4) and medium-concentrated ((**c**) *C*_Chit-g-PNIPAM_ = 0.5 mg/mL, pH = 3; (**d**) *C*_Chit-g-PNIPAM_ = 1 mg/mL, pH = 3) Chit-*g*-PNIPAM aqueous solutions as a function of shear rate (1/s) at different temperatures (25–50 °C) and pH values.

Moreover, the effect of the copolymer concentration, ionic strength (PBS) and pH of the medium on the rheological properties have been studied at different temperatures (Figure 2).

The shear viscosity (*η*) was registered as a function of the imposed shear rate from 1 to 100 s^−1^, in stationary continuous shear conditions, at different temperatures (25–50 °C). It was observed that, at a high concentration of copolymer (10 mg/mL in water, pH = 6), the viscosity drastically increased at temperatures situated around LCST, followed by a decrease in this property with a further increase in the temperature (Figure 2a). Therefore, the LCST transition could be considered a transition between dissolved copolymer chains and copolymer collapsed/aggregated chains, being characterized by a maximum number of inter-chain interactions. If the temperature is far below or far above the LCST of the copolymer, the solution viscosity is nearly constant with the applied shear rate (10–100 s^−1^), demonstrating that the copolymer solution has Newtonian behavior, while around LCST, the copolymer solution is non-Newtonian with a shear-thinning behavior. Thus, the LCST region could be considered as a reorganization temperature of polymeric chains due to the massive changing of chains conformation with the increase in the number/strength of hydrophobic interactions between isopropyl groups from different PNIPAM side chains of the Chit-*g*-PNIPAM graft copolymer. Using the same concentration of Chit-*g*-PNIPAM (10 mg/mL) but in PBS solution, all viscosity curves are situated at lower values (Figure 2b), as compared to the copolymer aqueous solution. Nevertheless, the same solution behavior has been recorded: i) increasing the viscosity with temperature, when T < LCST, and ii) decreasing the viscosity with temperature, when T > LCST. This small variation in viscosity curves has been attributed to the higher ionic strength generated by PBS, which could act as a “lubricant” between copolymer chains, with the intra-chain hydrophobic interactions being favored as compared to the inter-chain interactions. The disappearance or drastic reduction of the Lower Critical Solution Temperature (LCST) in Figure 2b when using PBS is likely due to the buffer’s influence on the polymer’s solubility based on a balance between electrostatic repulsive interactions and attractive hydrophobic ones. The buffer can screen the electrostatic interactions that drive phase separation due to the hydrophobic interactions. In Figure 2b, the temperature parameter will have a reduced effect, because the Chit-*g*-PNIPAM is already phase separated before the temperature is increased. Even if there is a phase separation induced by the salt from PBS, the formed aggregates will not affect drastically the viscosity of the suspension; at high shear rate, a Newtonian behavior is observed. In conclusion, Chit-*g*-PNIPAM copolymer in PBS suffers a partial phase separation at room temperature, and the subsequent increase in temperature will have a very low effect on the already aggregated chains.

At a lower concentration of copolymer in aqueous solution (1 mg/mL and 0.5 mg/mL) and in acidic pH (pH = 3, Figure 2c,d), the dynamic viscosity slightly decreases with the shear rate, with a low shear rate at (1–10 s^−1^) and constant at a high shear rate (10–100 s^−1^), demonstrating that some inter-chain interactions could exist at short distance. The behavior of viscosity as a function of the temperature showed a very low variance with the increase in the shear rate, due to the low concentration of copolymer chains in solution, which is correlated with very low number of inter-chain interactions.

**Figure 3:**
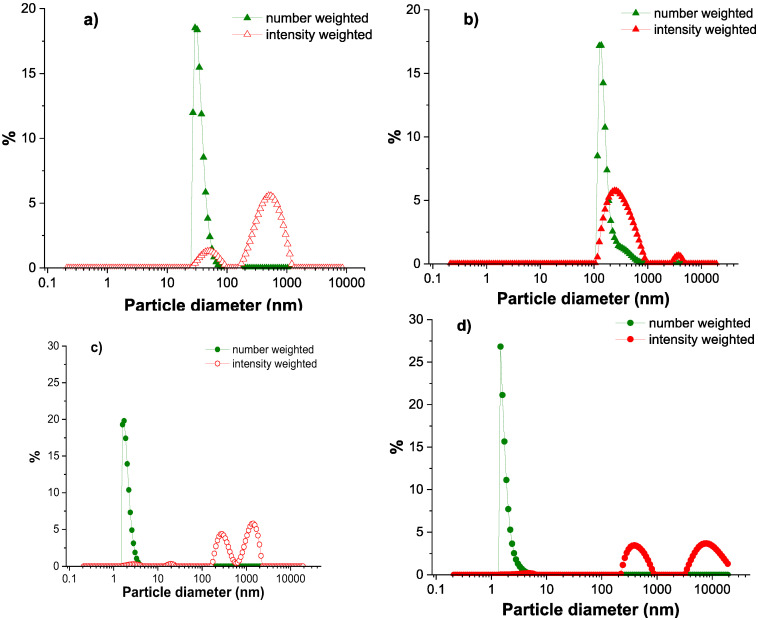
Diameter of particles estimated from DLS number and intensity size distributions for Chit-*g*-PNIPAM (**a**,**b**) and HSA (**c**,**d**), at 25 °C (**a**,**c**) and 37 °C (**b**,**d**) (*C*_Chit-g-PNIPAM_ = *C*_HSA_ = 1 mg/mL).

The binding process of negatively charged proteins, such as albumins, to positively charged polymeric chains, such as the modified chitosan utilized in this study, displaces counterions and water molecules from both types of macromolecules, leading to an increase in entropy, which favors the complex formation. Due to the versatility in the modified polysaccharides, the Chit-*g*-PNIPAM copolymer can be potentially utilized for the successful transportation of different types of proteins/enzymes cargoes, since for this type of application, it is very important to have a polymeric carrier that could offer stability, protection from the harsh media, and the ability to aim at specific cells and stimulate cellular entry. Before mixing, each individual aqueous solution was characterized by DLS measurements, where the size number/intensity distribution was followed at two temperatures (Figure 3).

The average hydrodynamic diameter of the copolymer chains, measured from the number weighted size distribution, increased from ~ 40 nm at 25 °C to approximately 200 nm at 37 °C, due to the PNIPAM side chains hydrophobic interactions (Figure 3a,b), while in the case of the HSA macromolecules, it was observed that the size was not influenced by the increase in temperature, with the hydrodynamic diameter being constant, below 10 nm.

**Figure 4:**
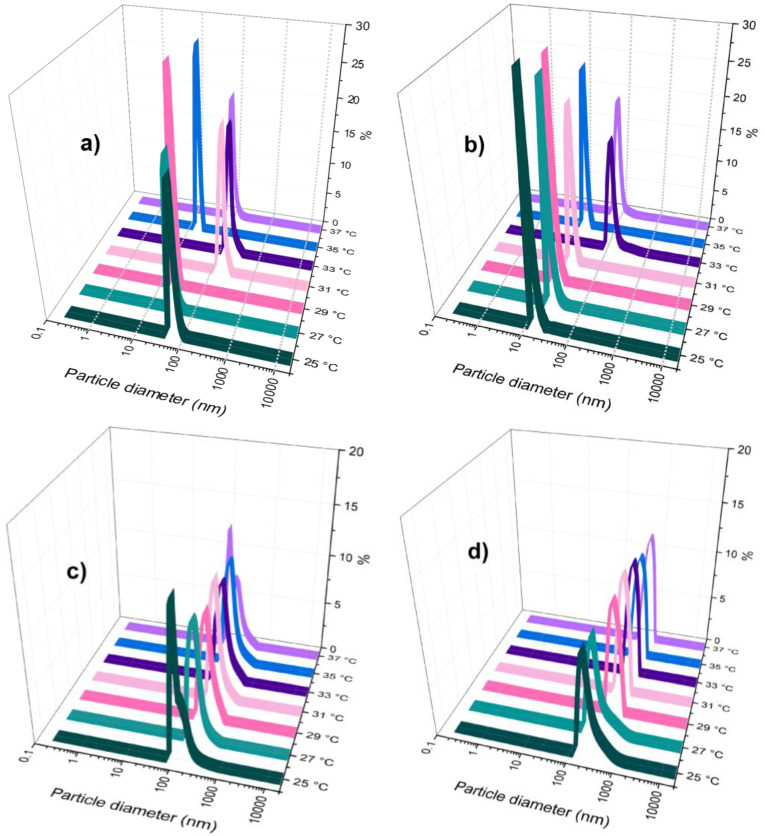
DLS number-weighted size distributions (%) of Chit-*g*-PNIPAM/HSA (nano)structures obtained at different molar ratios: [Chit-*g*-PNIPAM]:[HSA] = 5:2 (**a**), 5:5 (**b**), 5:10 (**c**), and 5:15 (**d**) as a function of temperature (25–37 °C).

**Figure 5:**
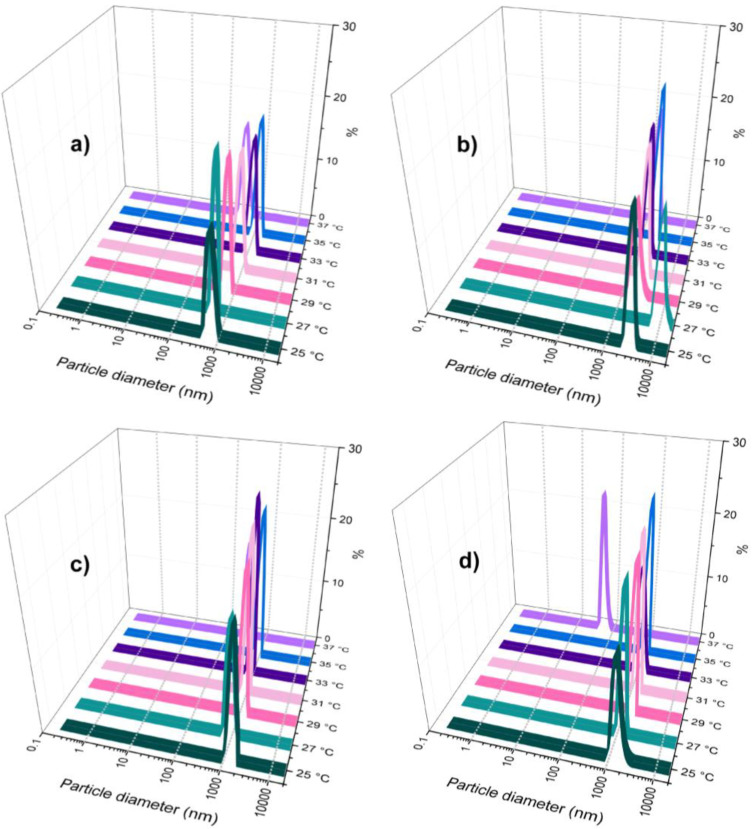
DLS number-weighted size distributions (%) of HSA/Chit-*g*-PNIPAM self-assembled microstructures obtained at different molar ratios: [HSA]:[Chit-*g*-PNIPAM] = 100:2 (**a**), 100:10 (**b**), 100:30 (**c**) and 100:50 (**d**), as a function of temperature (25–37 °C).

Based on the molecular sizes as a function of temperature for each component in aqueous solution without pH adjustment and at very low ionic strength, the non-stoichiometric interpolyelectrolyte protein/copolymer complex formation has been investigated by DLS measurements taking into account the order of addition of components and temperature (Figure 4, Figure 5 and Figures S2 and S3, Supplementary Materials).

In the first series of complex self-assembled structures, in the aqueous solution of the larger in size component (Chit-*g*-PNIPAM), whose concentration in molar parts was kept constant, the lower sized component, HSA, was added dropwise at different molar ratios, 5:2; 5:5; 5:10 and 5:15. At a low concentration of HSA (Figure 4a,b), the number size distribution as a function of the temperature showed that the Chit-*g*-PNIPAM/HSA self-assembled structures presented thermoresponsive properties, around 31–33 °C, demonstrating that the major component, at high molar ratio, dictated the thermoresponsiveness of the protein/copolymer complex self-assembled nanostructures. At LCST, the nanostructures size exceeded 100 nm, followed by a small decrease in size at higher temperature, presumably due to the further collapse (shrinking) of the formed macromolecular structures. By increasing the HSA concentration in the system (Figure 4c), it was observed that temperature does not have any relevant influence on the size of the Chit-*g*-PNIPAM/HSA nanostructures, and the diameter average plateau value for the complexes was observed at about 200 nm. This value was observed also for the initial Chit-*g*-PNIPAM chains at 37 °C (Figure 3b). Thus, the size of Chit-*g*-PNIPAM chain aggregates formed above LCST had similar size with the Chit-*g*-PNIPAM/HSA self-assembled nanostructures (Figure 4c), where temperature had no noticeable effect on the nanostructures’ size. Further, the [Chit-*g*-PNIPAM]:[HSA] = 5:15, showed very low size changes; even when the temperature increased from 25 to 37 °C, the nanostructures were stable around 300 nm.

To investigate the influence of the addition order of the components onto the complexe size, a second series of complexes was prepared in aqueous solution, with the HSA concentration being kept constant and Chit-*g*-PNIPAM being added dropwise. The DLS number size distribution was also recorded as a function of temperature (25–37 °C) (Figure 5).

**Figure 6:**
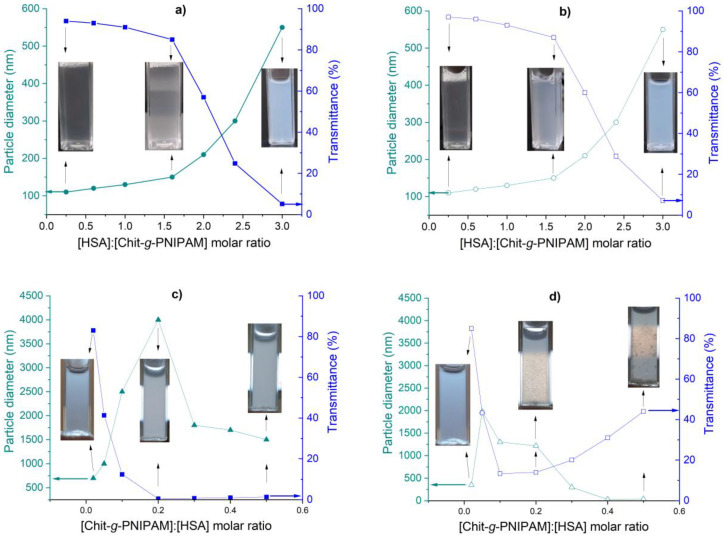
Particle diameter and dispersion transmittance determined simultaneously during DLS measurements for Chit-*g*-PNIPAM/HSA (**a**,**b**) and HSA/Chit-*g*-PNIPAM (**c**,**d**) self-assembled structures, obtained at different molar ratio of components and measured after 5 min (**a**,**c**) and 3 h (**b**,**d**) from initial mixing time (T = 37 °C, C_Chit-g-PNIPAM_ = C_HSA_ = 1 mg/mL).

It was observed that self-assembled structures of large size (>800 nm) were formed right at the beginning of copolymer solution addition to the protein solution (Figure 5a). This fact demonstrates that the copolymer chain, with polyampholyte character, shows a strong capacity for binding multiple protein globules based on electrostatics, hydrogen bonds, and hydrophobic interactions. A small increase in Chit-*g*-PNIPAM generated larger aggregates of self-assembled macromolecules, which exceeded 2000 nm (Figure 5b). Therefore, the semi-rigid Chit-*g*-PNIPAM chains seem to act like a flocculant for protein globules, where a small number of chains could bind a large number of HSA molecules, leading to a strong phase separation in time after solution mixing, where larger aggregates can sediment. Reaching the half of stoichiometry ([HSA]:[Chit-*g*-PNIPAM] = 2:1) between the two components, the size of the aggregates decreased to a value of 1000 nm, due to the redistribution of HSA macromolecules along the Chit-*g*-PNIPAM chains (Figure 5c,d). Moreover, the continuous aggregation/flocculation processes, which occur in time, led to the sedimentation of the complexes with larger sizes, allowing for the smaller sized complexes to be more “visible” in DLS measurements. This behavior is determined by the detection mode of the DLS technique, where the intensity fluctuations of scattered light are proportional with the hydrodynamic radius to the sixth power. Thus, at the beginning of the experiment, the larger particles dominate the signal and swamp the signal of the smaller ones. Then, when the large particles precipitate, their large intensity contribution vanishes, and the remaining signal becoming governed by the smaller complex particles, which were always there but screened in DLS detection by the larger ones. The size, concentration and stability of the formed self-assembled structures have been followed by DLS measurements as a function of the molar ratio between components (Figure 6).

In the first series, where Chit-*g*-PNIPAM was the major component in terms of the molar ratio, the size of the formed Chit-*g*-PNIPAM/HSA self-assembled nanostructures increased with the increasing HSA molar ratio. Moreover, this increase in complex size has been accompanied by a decrease in transmittance, which demonstrates the formation of self-assembled nanostructures at higher HSA content (Figure 6a). Thus, the formed nanostructures were stable in time at 37 °C; as can be seen comparing Figure 6a,b, the sizes of the dispersed nanostructures and solution transmittance remained constant during a period of 180 min. In the second series, the major component was HSA, and the self-assembled structures were formed by the progressive addition of Chit-*g*-PNIPAM solution. In this case, HSA/Chit-*g*-PNIPAM self-assembled structures were formed with large size even at very low concentration of the copolymer ([HSA]:[Chit-*g*-PNIPAM] = 100:2). This fact could be attributed to the high affinity of the semi-rigid stretched chains of the modified chitosan toward to the polyampholyte-type HSA protein (Figure 6c). Further increasing the amount of Chit-*g*-PNIPAM introduced in the system, a maximum in size (~4000 nm) was observed for the HSA/Chit-*g*-PNIPAM self-assembled structures at molar ratio [HSA]:[Chit-*g*-PNIPAM] = 100:20, followed by a decrease in size due to the appearance of the separation process at higher Chit-*g*-PNIPAM concentrations (Figure 6c). The process of phase separation in the gravitational field is more pronounced in time and takes place at a higher Chit-*g*-PNIPAM amount, as can be seen by a decrease in size and an increase in transmittance (Figure 6d). All the described processes—self-assembled structures formation, aggregation and sedimentation—occur before reaching the equimolar ratio of the components (i.e., [HSA]:[Chit-*g*-PNIPAM] < 100:50). Thus, the second series of complexes demonstrated that a low amount of copolymer introduced in HSA solution could interact and form large HSA/Chit-*g*-PNIPAM aggregates, which can sediment within a few hours.

**Figure 7:**
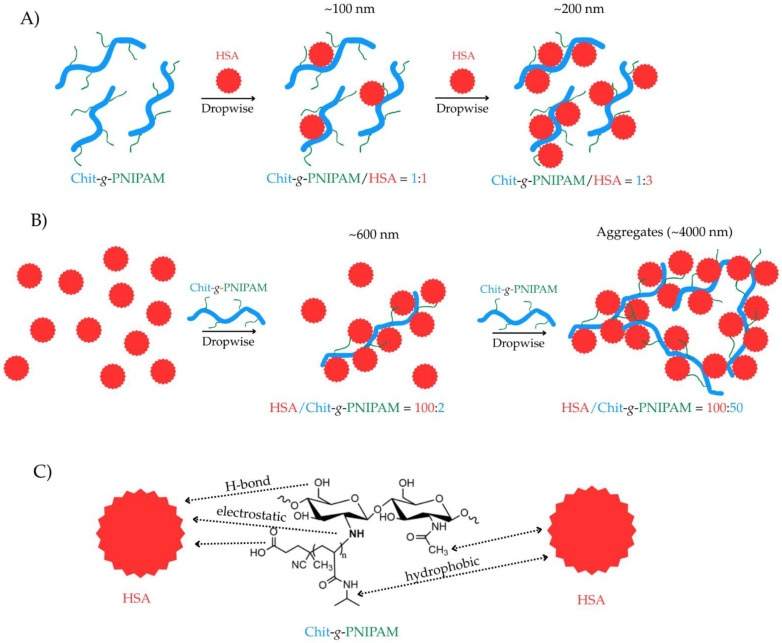
Schematics of complexes’ formation via different mixing protocols: Chit-*g*-PNIPAM/HSA (**A**) and HSA/Chit-*g*-PNIPAM (**B**), based on mutual interactions of chains (**C**), molar ratio and order of components addition.

In this study, it was demonstrated that the order of the component addition had a significant influence on the stoichiometry of the complexes, as well as on the time stability of the formed entities. At the concentration of components equal to 1 mg/mL, it is hard to determine the exact stoichiometry of the Chit-*g*-PNIPAM/HSA and HSA/Chit-*g*-PNIPAM self-assembled structures due to the multiple processes (formation by mutual interactions, aggregation by flocculation and continuous particle fractionation by sedimentation), which are present in the aqueous systems. Moreover, it can be stated that the Chit-*g*-PNIPAM copolymer chain size and conformation influenced the binding capacity toward HSA macromolecules; thus, one copolymer chain could establish more binding points with more HSA macromolecules compared with one HSA macromolecule, which bound fewer Chit-g-PNIPAM chains. Based on these results, a probable mechanism of the self-assembled structures formation between HSA protein and Chit-*g*-PNIPAM copolymer via mainly electrostatic/H-bonding/hydrophobic interactions is presented in Figure 7.

**Figure 8:**
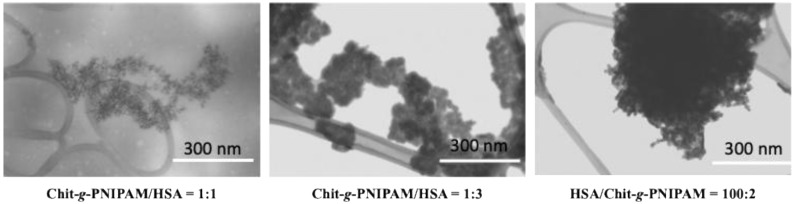
STEM images of the different types of Chit-*g*-PNIPAM/HSA and HSA/Chit-*g*-PNIPAM self-assembled structures obtained at different macromolecular molar ratios (scale bar: 300 nm).

An important parameter in designing controlled drug delivery systems is the interactions between components in the non-stoichiometric ratios. It is already known that by mixing two oppositely weakly charged polyelectrolytes at an increased concentration, a fluid complex coacervate is formed [55]. During coacervate formation, the polymeric chains densify by repelling water in hydrophobic micro-domains through the formation of physical cross-links. The components in the coacervates adhere strongly to one another, due to both the mechanical mixing and intra-chain charge overcompensation, acting like a glue. The final obtained material is held together by non-covalent ionic/H-bond and hydrophobic interactions with different bond strengths. These bonds are reversible and depend strongly on the solution conditions, such as the pH, ionic strength, polymer concentration and mixing ratio. The primary interactions are often driven by a combination of electrostatic and weak forces like hydrogen bonds and hydrophobic interactions, leading to the formation of an interpolyelectrolyte complex. Chitosan is a weak polybase, meaning its charge density is highly dependent on the pH. The protonation of chitosan’s amine groups in acidic pH is highly relevant in biological systems, especially in the context of drug delivery systems and specific physiological environments. The protonated amine groups (positively charged) are crucial for strong electrostatic interactions with the negatively charged regions of HSA, with these electrostatic interactions as the primary driving forces of the binding between HSA and Chit-*g*-PNIPAM, especially at acidic pH, with different biomedical applications. For example, the highly acidic environment of the stomach (pH 1.5–3.5) causes full protonation of Chit-*g*-PNIPAM, leading to increased solubility and potential interaction with HSA or drug release in this area. Another example could be tumor environments, which are often more acidic than normal physiological conditions (blood pH ~7.4). Chit-g-PNIPAM can remain stable and encapsulate drugs at physiological pH, but then it may swell, change properties, and release the drug in the acidic tumor environment. In addition, variations in the molar ratio of components can alter the overall hydrophobicity or hydrophilicity of the modified chitosan, which can influence the interaction with HSA. The present study aims to establish a link between the modified chitosan’s properties, which are affected by numerous parameters (pH, ionic strength, and ratios of polymeric chains), and its subsequent interaction with HSA. In the first series, the non-stoichiometric Chit-*g*-PNIPAM/HSA self-assembled nanostructures are formed by the progressive retention of HSA protein molecules by individual Chit-*g*-PNIPAM chains, with the size of the nanostructures increasing step-by-step with the addition of HSA macromolecules. At the molar ratio [Chit-*g*-PNIPAM]:[HSA] = 1:3, the self-assembled nanostructures are stable in time, with a size around 200 nm (Figure 7A). In the second series, the non-stoichiometric HSA/Chit-*g*-PNIPAM complex nano-agglomerates are formed in large sizes (~600 nm) at a very low content of copolymer, due to the high affinity of the polymeric chains toward protein macromolecules. During the further increase of Chit-*g*-PNIPAM concentration in the aqueous system, the newly introduced chains act like a macromolecular cross-linker, with the already formed HSA/Chit-*g*-PNIPAM self-assembled structures, producing large aggregates and flocs with various sizes, exceeding micrometer sizes. The stability of the formed self-assembled structures in the second series was very low, with a large amount of sediments/coacervates observed. Thus, the second approach seems more suitable for the formation of large polymeric networks based on proteins and polymers, like gels, also with potential applications in the creation of new other types of soft hybrid materials. The formation of different self-assembled (nano)structures depending on the order of addition and the macromolecular molar ratio can be seen in the STEM images (Figure 8).

In the first series, small nanostructures (100–200 nm) were obtained (Figure 8, Chit-g-PNIPAM/HSA = 1:1), while in the second series, larger aggregates were observed (> 500 nm) (Figure 8, HSA/Chit-g-PNIPAM). The STEM images support the proposed mechanism of Chit-*g*-PNIPAM and HSA complex formation dependent on the macromolecular molar ratio and order of addition, as shown in Figure 7. The grafted PNIPAM chains onto chitosan modulate the intermolecular interactions with HSA and contribute to temperature-dependent structural rearrangements, even if a sharp LCST-type transition is not evident at a high content of HSA in the complex, as clearly evidenced in Figure 4 and Figure 5. Nevertheless, the study demonstrated that the HSA content (controlled by the molar ratio of components) as well as the order of addition of components drastically influenced the size and thermoresponsiveness of the system. The presence of PNIPAM on a chitosan backbone alters the hydration properties, steric stabilization, and hydrophobic interactions within the complexes, which may influence their structural organization and potential application performance.

## 4. Conclusions

The Chit-*g*-PNIPAM graft copolymer and the model protein HSA have been successfully utilized to obtain non-stoichiometric protein/copolymer self-assembled structures. The aqueous solutions of individual components showed the thermoresponsive behavior of the graft copolymer due to the PNIPAM side chains, with a phase separation at around 31–33 °C, as determined by turbidimetric and light scattering methods. Moreover, the rheological parameters were strongly influenced by the copolymer LCST value, with an increase in viscosity before LCST followed by a decrease after LCST. By changing the molar ratio and the order of addition of Chit-*g*-PNIPAM and HSA (5:2 to 5:15) or HSA and Chit-*g*-PNIPAM (100:2 to 100:50), two series of Chit-*g*-PNIPAM/HSA and HSA/Chit-*g*-PNIPAM self-assembled structures have been obtained, respectively. By mixing the two types of macromolecular chains, it was demonstrated that the order of addition of components affected the size and stability of the formed self-assembled structures. In the first series, Chit-*g*-PNIPAM/HSA, where the Chit-*g*-PNIPAM copolymer concentration was kept constant, and the HSA concentration increased step by step, sizes of hundreds of nanometers were obtained, both before and after the point of stoichiometry, while for HSA/Chit-*g*-PNIPAM, where the HSA concentration was kept constant, microstructures were formed, even at a very low concentration of Chit-*g*-PNIPAM. In the second series, the formed self-assembled structures were unstable and sedimented during a period of 10 h. By this study, we have shown the main advantages of our system: (1) the dual interaction mechanisms (electrostatic and thermally induced hydrophobic interactions) influenced/controlled by the pH of the environment; (2) the tunability of the particles size via temperature adjustment and molar ratio of mixed components; (3) the reversible assembly/disassembly behavior of formed (nano)structures at low content of HSA molecules. Therefore, the interactions studies of the Chit-*g*-PNIPAM with HSA are of great importance for the potential applications of such materials in designing organic architectures for the fields of controlled drug delivery, biomimetics and biomaterials development.

## Data Availability

The original contributions presented in this study are included in the article or Supplementary Materials. Further inquiries can be directed to the corresponding author.

## Supplementary Materials

The following supporting information can be downloaded at https://www.mdpi.com/article/10.3390/polym18040515/s1, Figure S1. LCST determination of Chit-*g*-PNIPAM copolymer using DLS measurements (intensity weighted size distributions); Figure S2. DLS intensity weighted size distributions (%) of Chit-*g*-PNIPAM/HSA self-assembled structures obtained at different molar ratios: [Chit-*g*-PNIPAM]:[HSA] = 5:2 (a), 5:5 (b), 5:10 (c) and 5:15 (d), as a function of temperature (25–37 °C); Figure S3. DLS intensity size distributions (%) of HSA/Chit-*g*-PNIPAM self-assembled structures obtained at different molar ratios: [HSA]:[Chit-*g*-PNIPAM] = 100:2 (a), 100:10 (b), 100:30 (c) and 100:50 (d), as a function of temperature (25–37 °C).
