# Supplementary material for: Self-Assembled (Nano)Structures of Human Serum Albumin with Thermoresponsive Chitosan-g-PNIPAM Graft Copolymer
Source: Polymers (Basel). 2026 Feb 19;18(4):515. doi: 10.3390/polym18040515 (PMC12944366; doi:10.3390/polym18040515)
Supplement: Supplementary file 1 [file polymers-18-00515-s001.zip › polymers-4164606-supplementary.pdf]

Supplementary information's

Self-assembled (nano)structures of human serum albumin with thermoresponsive Chitosan-*g*-PNIPAM graft copolymer

Florin Bucatariu[1], Larisa-Maria Petrila[1], Timeea-Anastasia Ciobanu[1], Marius-Mihai Zaharia[1], Stergios Pispas[1,2], Marcela Mihai[1,*]

[1] Petru Poni Institute of Macromolecular Chemistry, 41A Grigore Ghica Voda Alley, 700487 Iasi, Romania; fbucatariu@icmpp.ro; larisa.petrila@icmpp.ro; timeea.ciobanu@icmpp.ro; zaharia.marius@icmpp.ro; marcela.mihai@icmpp.ro.

[2] Theoretical and Physical Chemistry Institute, National Hellenic Research Foundation, 48 Vassileos Constantinou Ave., 116 35 Athens, Greece; pispas@eie.gr

* Correspondence: marcela.mihai@icmpp.ro

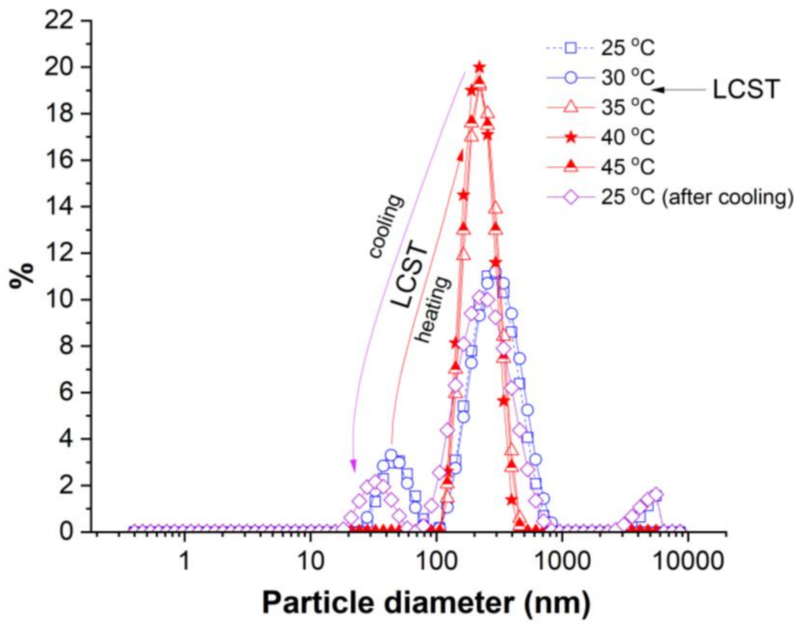

Figure S1. LCST determination of Chit-*g*-PNIPAM copolymer using DLS measurements (intensity weighted size distributions).

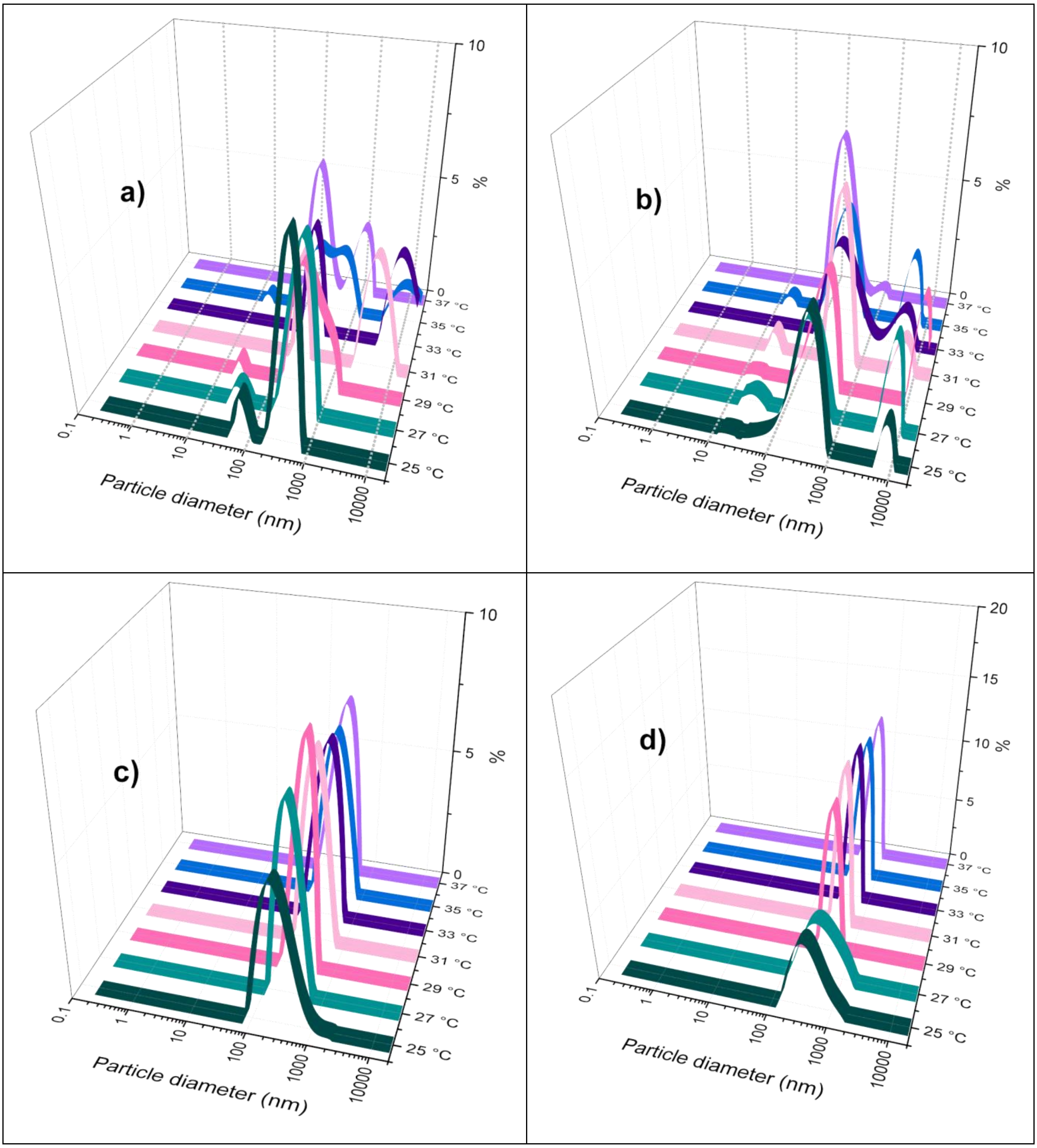

Figure S2. DLS intensity weighted size distributions (%) of Chit-*g*-PNIPAM/HSA self-assembled structures obtained at different molar ratios: [Chit-*g*-PNIPAM]:[HSA] = 5:2 (a), 5:5 (b), 5:10 (c) and 5:15 (d) in function of temperature (25 – 37 °C).

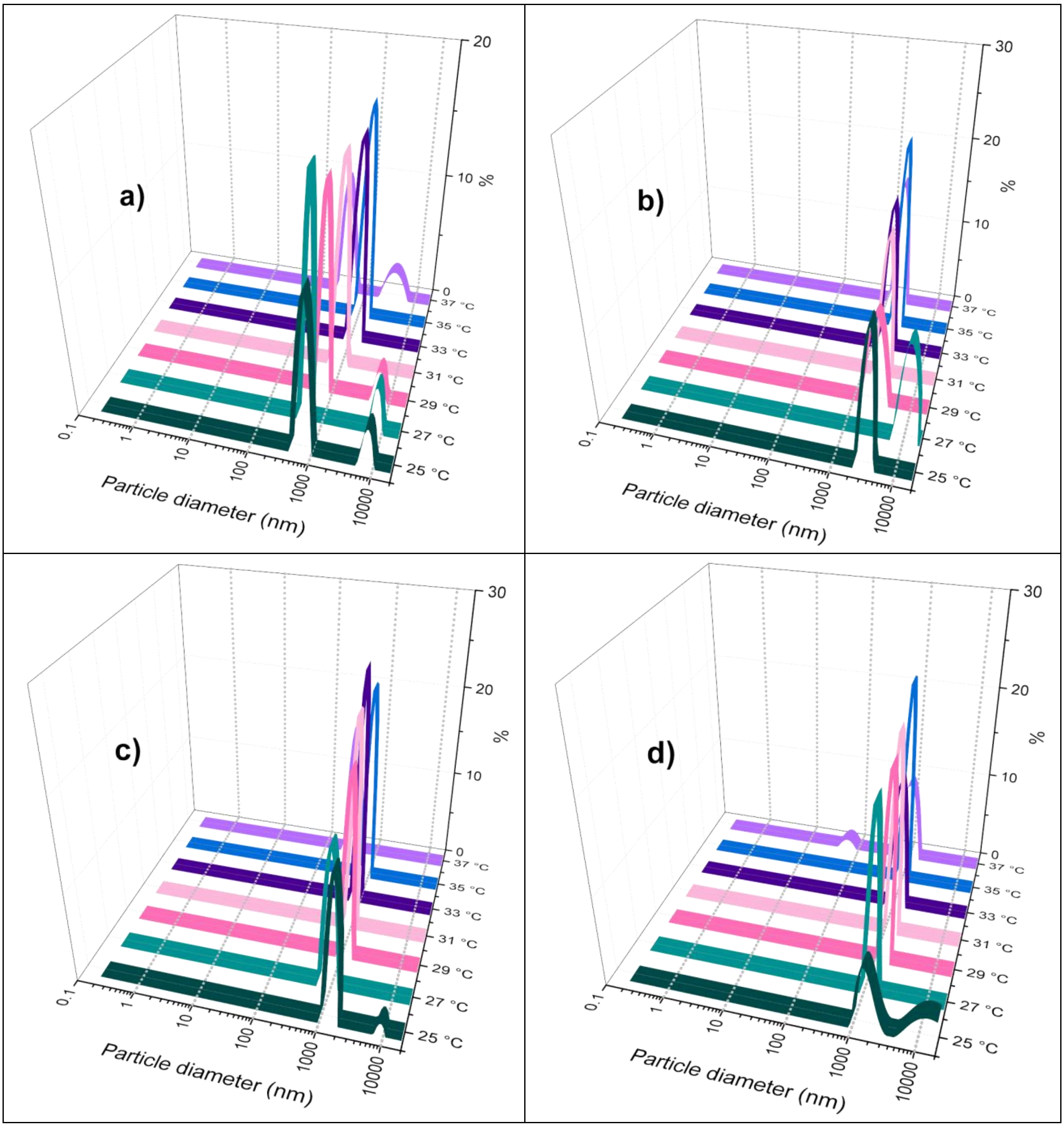

Figure S3. DLS intensity size distributions (%) of HSA/Chit-*g*-PNIPAM complexes obtained at different molar ratios: [HSA]:[Chit-*g*-PNIPAM] = 100:2 (a), 100:10 (b), 100:30 (c) and 100:50 (d) in function of temperature (25 – 37 ^{o}C).
